# The SUPA mechanisms of crime opportunity evolution

**DOI:** 10.1186/s40163-026-00300-1

**Published:** 2026-10-24

**Authors:** Daniel Hammocks, Kate Bowers, Bennett Kleinberg, Shane D. Johnson

**Affiliations:** 1 Dawes Centre for Future Crime at UCL, Department of Security and Crime Science, University College London, London, UK; 2 Data Science & Insights Team, Mayor’s Office for Policing and Crime, London, UK; 3 Department of Security and Crime Science, University College London, London, UK; 4 Department of Methodology and Statistics, Tilburg University, Tilburg, Netherlands

**Keywords:** Crime opportunity, Evolution, Modus operandi, Horizon scanning, Future crime, SUPA

## Abstract

The nature of crime is changing, and so are the opportunities that enable it. Fifty years since the seminal work on crime as opportunity, we suggest that there is a need to consider ways to measure how and why opportunities have evolved. Specifically, we propose four mechanisms of crime opportunity evolution, inspired by biological speciation theory and adapted here to account for the cultural transmission of criminal behaviour, with evolution measured through changes in modus operandi. These are the SUPA mechanisms: Space-Driven (allopatric), Utility-Motivated (sympatric), Pressure-Driven (peripatric), and Attraction-Led (parapatric). We connect these mechanisms of evolution to crime opportunity theory and situational crime prevention. Space-driven opportunities arise through distinct geographic contexts; utility-motivated as a result of success (through choice or chance) within the same opportunity context (often made possible by new tools or technologies); pressure-driven evolution through the blocking of prior crime context-mechanism combinations; and, attraction-led evolution through a voluntary change in context. We illustrate each mechanism with historical examples, discuss how we might best prepare for each type, and consider how the SUPA mechanisms might apply to horizon scanning and future crime prevention practice.

## Introduction

Crime is dynamic and importantly, so is crime opportunity (for the seminal work on crime as opportunity see Mayhew et al. 1976). Consequently, the intelligence, systems and procedures used by those who wish to detect and prevent crime need to adapt constantly. The typically exponential evolution and adoption of technology, along with societal change, make anticipating and responding to *changes* in crime opportunity critical if we are to prevent the emergence of new problems, or to prevent them from becoming established. To do this, we need to understand what ‘newness’ is and where to look for it.

The aim of better understanding crime evolution aligns with the ideas of Ekblom (2013) who describes crime as "innovative and surprising", with offenders continually seeking to capitalise on the various situations and opportunities presented to them by constantly adapting and bettering their methods of perpetration (Ekblom, 1997). A key technique for anticipating change, often used in the business domain (Aguilar, 1967) is Environmental or Horizon Scanning (HS). HS is a process whereby the external environment is scanned for signals of future change, both weak and strong (Ansoff, 1975), that provide insight about the future with varying levels of certainty. HS is based on the assumption that change is rarely spontaneous but instead emerges over time (Ferguson, 1993), with weak signals typically appearing first in (say) fringe or scientific literature before surfacing in mainstream sources (Hiltunen, 2008; Molitor, 2003). If such early signals are identified they help to anticipate potential future change, increasing the time available to mitigate and prevent negative consequences. In essence, this should help provide law enforcement agencies with early warnings about possible future threats (Johnson et al., 2018; Johnson, 2024; Stanley et al., 2015).

For crime, early signals of future change may be detected through the emergence of new modus operandi (MO) which describe the way crimes are committed (Ekblom, 2002). However, existing techniques of scanning for future crime opportunities tend to focus on statistical trends and neglect the conceptual underpinnings of why or (most pertinent to this paper) how change occurs. It is also important to clarify that future crime and future crime opportunities are not synonymous. Eck et al. (2026) (this volume) conjecture that opportunities can exist before the realisation of a crime incident. A number of factors will determine whether offenders realise these opportunities resulting in crime. These include social or cultural factors (e.g. communication channels), whether offenders have the appropriate resources for crime (see Ekblom and Tilley 2000), and whether novel behaviours are recognised as criminal in the first place.

Moreover, in relation to crime, HS is challenging as it seeks to identify both new ways of committing crime events (e.g., the theft of cars through intercepting signals from nearby key fobs (Brown et al., 2025)) as well as new forms of offending that are yet to be criminalised (e.g., the generation of non consensual intimate images using AI (Hawkins et al., 2025)).^1^

Whilst different approaches to defining crime exist, this paper adopts the legal perspective and proposes that *completely new crime types very rarely materialise*. Instead, what changes is not the legal category of offence (e.g., theft, fraud, assault) but the crime opportunities available and the methods by which they are exploited – the MO. Consequently, larger scale trends in crime mostly occur as new methods of perpetrating *existing* legally-defined offences more easily or at greater scale emerge. These new methods are shaped by changes in the available mechanisms and contexts of crime opportunities. The terms *mechanism* and *context* are used deliberately here. Pawson and Tilley (1997) propose that crime prevention measures work according to context-mechanism-outcome combinations (CMOCs). For example, CCTV will work better in specific ways in certain situations. To elaborate, CCTV mechanisms might include the deterrence that overt cameras afford, or the detection of offenders through collected footage. Contexts might include car parks or underpasses on residential estates. The most promising mechanisms for prevention often differ across different contexts leading to CMOCs that are successful in terms of their aspirational preventative outcome and those that are not. Analogously, these ideas also apply in reverse to *offending*. That is, there will be particular contexts that are conducive to particular mechanisms (or ways) of offending that can be employed for specific types of crime. Indeed, situational prevention works by blocking these and is a key reason why Ron Clarke argued that preventers need to be *crime-specific* (Clarke, 1983, 1997). Building on this idea, we need to consider (1) potential new contexts (or opportunity creation settings) that are compatible with existing mechanisms that enable specific offences (e.g., a new poorly designed car may facilitate car theft, or lockdown restrictions may lead to an elevated risk of domestic abuse during a pandemic), (2) new exploitation mechanisms that could emerge (e.g. through the misuse of new technologies, offenders learning new methods of exploitation, or through new “crime-as-a-service” offers (Manky, 2013)) and (3) interactions between the two.

The aim of this paper is to marry up this take on crime opportunity evolution (consideration of new creation contexts and new exploitation mechanisms) from a crime science perspective to parallel ideas of adaptation from the literature that focuses on biological evolution. That is, we apply environment-based evolution ideas from the natural world to the environment-driven evolution of the cultural problem of crime. In doing so we aim to develop a way of classifying and understanding *different types* of crime evolution. In turn, this typology should help with systematic HS and future foresight exercises concerned with future crime and crime opportunities.

The remainder of this paper begins by laying out our working definitions of terms used; crime, future crime, crime opportunity and MO. We then introduce the biologically inspired crime opportunity evolution framework and use examples to explore its potential application. Finally, we discuss the strengths and limitations of the proposed approach.

## Crime, future crime, modus operandi and crime opportunity

### Crime and future crime

What is crime? Various definitions exist; here we take the legal definition, summarised by Turner (2013) as an act meeting at least one of three characteristics: (a) causing a harm, brought about by human conduct, which the sovereign power in the State desires to prevent; (b) that among the measures of prevention selected is the threat of punishment; and (c) for which legal proceedings of a special kind are employed to decide whether the person accused did in fact cause the harm, and is, according to law, to be held legally punishable for doing so.

A limitation of the legal definition of crime is that it is retrospective (Brodeur & Ouellet, 2005). That is, an act must be known about and prevalent before it can become criminalised, conditions that entirely new forms of crime cannot meet. However, since our framework is intended to be practically useful to law enforcement, the remainder of this paper considers crime from the legal perspective and as measured and documented by law enforcement agencies. From this perspective, *future crime* is thus typically the significant emergence of a new method or way of committing crime that can be captured by current law enforcement or legal systems. It is, however, acknowledged that this might limit thinking on evolution over longer time horizons, for which alternative definitions of crime may be more appropriate. As noted earlier, there is a distinction between understanding *future crime* and understanding *future crime opportunity* which we will discuss again shortly.

### Modus operandi

The next stage in the identification of novelty is in the construction of a reliable framework that enables differentiation between crimes of the same legal type. One approach is through hierarchical classifications, such as that used by the UK Home Office. However, a more granular method particularly suited to HS is through analysis of the methods by which crimes are perpetrated — their MO.

Atcherley (1913) made the first known attempt at defining and recording offending behaviour with a focus on policing practice by developing a classification system which decomposed the MO of a crime into ten components, not all of which are necessarily present. Derivatives of Atcherley’s work are still used today, with learning material produced for new officers by the National Policing Improvement Agency (2012)^2^ describing MO using ten attributes that are remarkably similar to Atcherley’s, but organised into the mnemonic "STOP CRIMES". These attributes are listed in Appendix A, alongside Atcherley’s corresponding attributes in the second column. Moreover, the national crime recording standard in the UK stipulates that MO must be included in police crime reports (College of Policing, 2019) which makes (some) data regarding them readily available.

A brief scoping review undertaken by Hammocks (2025) searched for academic literature on MO to see if it could add further to the definition. This suggested a contrast between the above practical definition, which follows a categorical classification system differentiating between specific attributes of an MO, and academic work which tends to lean towards broad definitions or none at all. We therefore suggest using the defined building blocks of the classification system to determine novelty in crime, whilst acknowledging that the classification system itself requires updating.

### Crime opportunity

This paper is about horizon scanning for the evolution of crime, but it is also about the evolution of crime opportunity, since changes to opportunity determine which crimes are possible. It is therefore important to distinguish between our understanding of the two. The concept of crime opportunity originates with Mayhew et al. (1976) and has since been examined by others, including Eck et al. (2026) in this special edition. They argue that opportunity has two elements; opportunity creation and opportunity exploitation by offenders.^3^ This can be seen as distinct from crime itself in that crime is the *realisation* of (a subset of) available opportunities. A useful framework through which to understand the process of the evolution of criminal activity would address both aspects (crime *and* crime opportunity). Law enforcement recording systems and data might be best suited to analyses of evolutions in *crime* by examining emerging MOs (through crime record analysis), whereas other methods of HS, including red-teaming, expert elicitation exercises and the scanning of weak and other signals, might be better suited to the consideration of the evolution of *potential crime opportunities* in the longer term. Emerging crime patterns indicate what has happened and is starting to gather momentum as a future crime trend, whereas the identification of future crime opportunities speaks to what might happen, should the conditions conducive to their exploitation arise. This is consistent with the three horizons model (Sharpe, 2015) used in futures work which differentiates between the current/near-, the medium-, and the long-term horizons. Whilst the first horizon is immediately visible, the others are not but the drivers of them will be to differing degrees and the aim of HS is to understand how they might influence the future operating environment.

## An evolutionary framework for understanding crime opportunity: the SUPA mechanisms

Whilst novelty evidently exists in the crime domain (e.g. when new legislation is passed) it can be argued, and shown, that in the majority of instances "new crimes" are the emergence of the same legal crime type exploited through a new MO, not necessarily the development of a new crime type (e.g. car thefts that exploit vulnerabilities in passive key entry systems are still car thefts, child sexual abuse material (CSAM) created by AI is still CSAM). We are seeking a framework that can not only identify different types of emerging crime opportunity, but also serve as the basis for futures exercises concerning how and why those opportunities arise. Where opportunities *are* realised as crime, MO provides the empirical unit through which that evolution can be observed.

We therefore draw on a theoretical/conceptual foundation from the study of evolution. Several evolutionary concepts could be considered relevant, but below we use a framework that deliberately borrows terminology from biological speciation and refines it for purpose. The rationale for using biological speciation is simply that opportunity theories of crime and crime science focus on the environmental and situational drivers of crime and see these as the most relevant to prevention. This aligns well with Darwinian evolution, for which the underlying principle is that it is *suitability to the environment* that drives biological evolution. That is, when environmental change occurs, adaptation takes place through the selection of biological mutations or existing genetic variants that are best suited to — or can best exploit — that environment; analogously, offenders may combine or improve existing elements of their criminal MO in new configurations, rather than devising entirely novel methods. Similarly, we suggest that methods of offending that are ill-suited to an environment are unlikely to persist, while those that are compatible with it are more likely to continue or to emerge. Consequently, if measuring realisation in terms of MO, it can be argued that *novelty is only relevant if it is sustainable*: short-lived deviations that do not persist are not evidence of opportunity evolution, whereas those that take hold and continue signal a genuine shift in the underlying opportunity structure.

However, it is important to be clear that criminal MO can and do evolve through cultural evolution – that is, the transmission of behaviours, techniques, and knowledge through social learning, imitation, and communication (Boyd & Richerson, 1988; Richerson & Boyd, 2006) – rather than through biological mechanisms alone (Ekblom, 2017). For crime, this distinction is critical: whilst biological evolution requires generations to manifest change, criminal innovations through cultural evolution can spread through networks (on or offline) at pace (Ekblom, 1997). Cultural transmission fundamentally speeds up the opportunity evolution process, enabling offenders to rapidly adopt successful methods developed by others and adapt to new opportunities or constraints.

Thus, the four mechanisms presented below, whilst based on a biological evolution framework, are adapted to describe cultural evolutionary processes, where crime opportunities evolve through rational choice, social learning, and environmental adaptation rather than genetic selection. This aligns with the notion that the particular constellation of opportunities, costs and benefits determines the "choice-structuring properties" of specific offences (Cornish, 1989).

Below, we use the general term ’mechanisms’ to refer to the pathways and conditions under which new crime opportunities arise, which should be (and is where necessary) distinguished from the context-mechanism-outcome combinations of Pawson and Tilley (1997) which describe how specific types of criminal act are prevented (and in this paper, enabled).

Drawing inspiration from ecology, we now redefine the four geographic modes of biological speciation – the evolution of a new distinct species (Schluter & Rambaut, 1341) – into four mechanisms through which new crime opportunities emerge. Based on the original work of Darwin (1872) on natural selection, Mayr (1942, 1954, 1963) categorises natural speciation into four modes:

${\boldsymbol{Allopatric}}$: “Speciation that occurs when [...] populations of the same species become isolated due to geographical” separation (Cabej, 2012).
${\boldsymbol{Sympatric}}$: “The splitting of one evolutionary lineage into two without the occurrence of geographic isolation” (Berlocher & Feder, 2002).
${\boldsymbol{Peripatric}}$: “[Speciation] in which a peripherally isolated population diverges to become a new species” (Losos & Glor, 2003).
${\boldsymbol{Parapatric}}$: The formation of a new species from populations which are geographically adjacent and therefore allow for restricted spatial interaction between the two populations; often considered a less extreme case of allopatric speciation (Mayr, 1963; Wu & Ting, 2004).

**Fig. 1:**
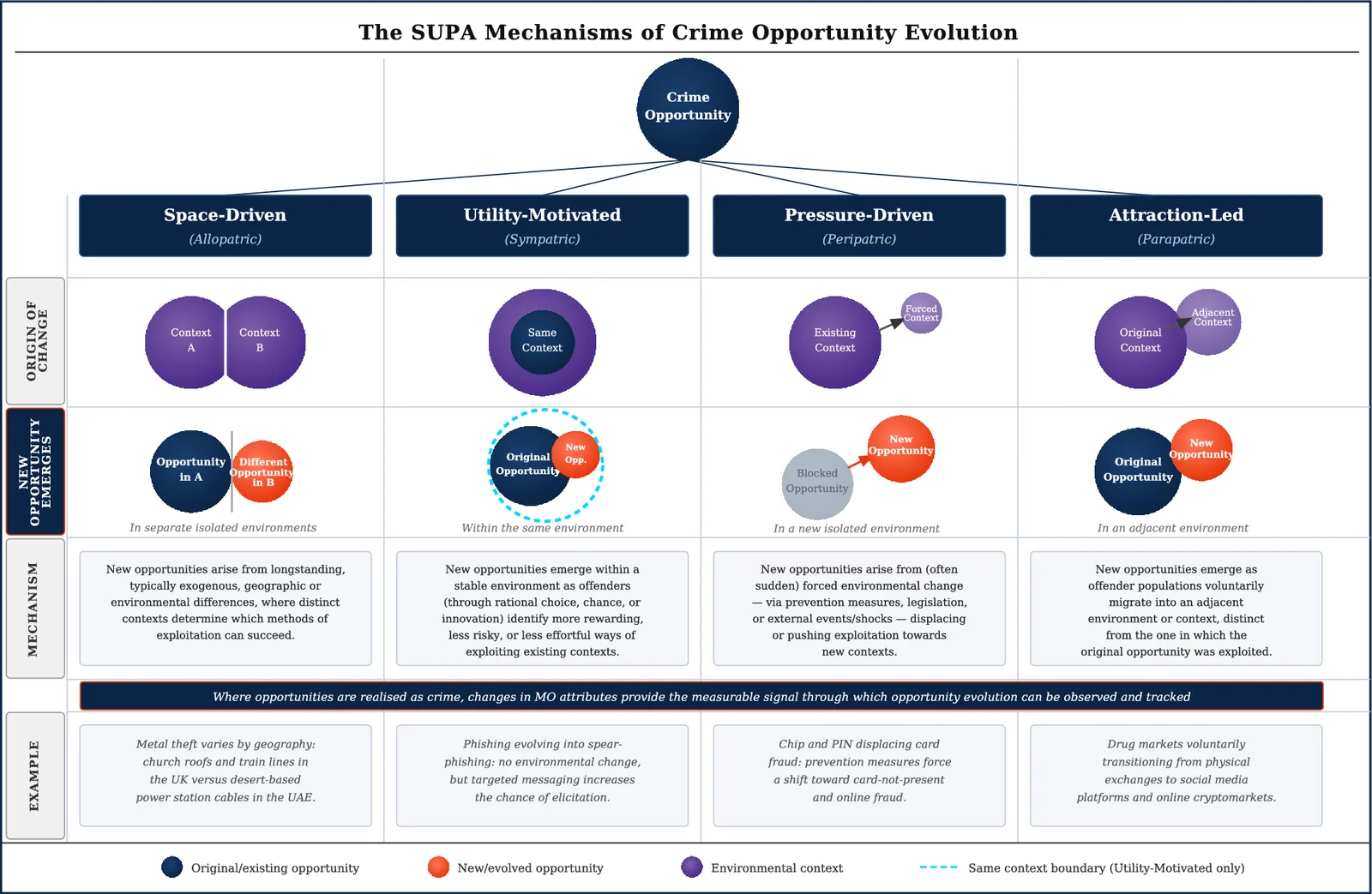
Diagram conveying The SUPA Mechanisms of Crime Opportunity Evolution: four pathways through which new crime opportunities emerge

The geographic foundation for each of these four ecological modes correlate with the notion that the occurrence of a crime event is in large part determined by the environment (Cohen & Felson, 1979), and hence the four ecological modes can be redefined into the Mechanisms of Crime Opportunity Evolution. In translating the ideas, an important consideration is that biological explanations discuss animal populations as the unit of analysis that may or may not adapt. In what follows, each mechanism describes a pathway through which new crime and crime opportunities can emerge (and also cease) with MO populations being the measurable unit of analysis by which that evolution can be tracked (for realised crime), rather than offender populations. Offenders execute the new MOs that realise those opportunities, but we are agnostic as to whether it is the same offenders or different ones executing them. To aid recall and practical application, we term these the Space, Utility, Pressure, and Attraction (SUPA) Mechanisms of Crime Opportunity Evolution. The four SUPA mechanisms are detailed below, alongside their biological counterparts, and visualised graphically in Fig. 1:

**Space-Driven (Allopatric)**: New crime opportunities arise from long-standing, typically exogenous, geographic or environmental differences, where distinct contexts determine which methods of exploitation can succeed.
**Utility-Motivated (Sympatric)**: New crime opportunities emerge within a stable environment as offenders – through rational choice, chance, or innovation – identify more rewarding, less risky, or less effortful ways of exploiting existing contexts (which may be facilitated by emerging technology or its adoption).
**Pressure-Driven (Peripatric)**: New crime opportunities arise from (often sudden) forced environmental change – through crime prevention measures, legislation, or significant external events/shocks – that displaces or pushes exploitation towards new contexts.
**Attraction-Led (Parapatric)**: New crime opportunities emerge as offender populations voluntarily migrate into an adjacent environment or context, distinct from the one in which the original opportunity was exploited.

We now test the applicability of these evolutionary definitions by (1) aligning them with concepts in crime science, (2) providing examples of where these might have occurred historically (i.e. have been realised) to produce novel MOs and (3) using them to describe crime opportunity evolution scenarios.

### Space-driven (allopatric)

The field of Crime Prevention through Environmental Design (CPTED) has identified how changes to built environments can reduce crime (Newman, 1972). For example, the type of crime opportunities available to an offender, and the methods by which they can be exploited, have been shown to vary based on the geographical and topographical features of environments (Felson, 2002). This provides circumstantial evidence for the space-driven mechanism; where distinct geographic/environmental contexts generate distinct crime opportunities, and the MOs through which they are exploited diverge accordingly.

While less likely to be observed over short time periods, and in environments with similar natural and built features, it can be espied as crime evolution over time in distinct environments. One example is how the opportunity for residential burglary, and the methods of exploitation it affords, varies by location. In Florida, where some properties are positioned adjacent to a river, offenders approach homes from the rear via boats (Freeman, 2009). In contrast, in suburban areas of the UK, wheelie bins have been used to aid access over (say) gates and fences (Ekblom, 2001). The crime type is the same; it is the environmental context that generates a different opportunity and, consequently, a different MO.

This idea is further reinforced by the Brantingham’s (Brantingham & Brantingham, 1995) work on the criminality of place and how "The ways in which we assemble [...] the urban backcloth can have enormous impact on [...] the quantities, types and timing of the crimes we suffer". In other words, environmental differences can directly affect available opportunities and the MO through which they are realised. Revisiting the idea of context-mechanism-outcome combinations from the offender exploitation viewpoint, in space-driven opportunity evolution, the context dictates which methods of exploitation are viable and most likely to produce a successful outcome (those that are “selected”).

In scanning for *future* crime opportunities we might imagine how future environmental (slow and steady) changes could generate new opportunity contexts in specific settings. For example, in a scenario where global warming leads to flooding in northern regions and extreme drought in southern ones, the opportunity contexts – and thus the methods of exploitation most likely to succeed – would differ markedly across those environments. Conversely, if environmental change causes one context (context A) to come to resemble another from which it previously differed (context B), we might anticipate that opportunities already present in context B – and the MOs through which they are realised – will emerge in context A for the first time. In this sense, examining crime opportunities in analogous environments can serve as a predictive tool: what exists in context B today may signal what is coming in context A tomorrow.

### Utility-motivated (sympatric)

Whilst the other SUPA mechanisms involve a change in the broader environment (e.g., geographic, regulatory, or contextual) the utility-motivated mechanism is distinctive in that the opportunity context itself remains stable. Instead, it is the *internal* environment that changes: new tools, technologies, or methods become available that alter the opportunity landscape within an existing context, enabling offenders to exploit it more effectively, at greater scale, or with reduced risk. This is not an absence of environmental change, but a different *type* of environmental change – one that operates *within rather than across* contexts.

This closely corresponds with the rational choice perspective and the idea that offenders look to reduce perceived risks and effort whilst maximising potential rewards (Clarke & Cornish, 1985), but the utility-motivated mechanism adds an important dimension: the role of learning and adaptation over time. Whilst rational choice explains what offenders optimise for, it does not fully account for how knowledge of more successful methods spreads across offending populations, or how the opportunity landscape itself shifts as new tools and technologies emerge. In striving to satisfy their success criteria, offenders are likely to seek, or stumble upon, alternative methods of exploitation. Where a more successful method is found, a transition may be observed (overall, it will be “selected” as an MO more often by exploiter populations) and potentially adopted more widely. Importantly, such evolution need not be triggered by external change. The stable context itself provides a space within which offenders can experiment and innovate, identifying new exploitable opportunities simply because the opportunity space permits it. Note that evolution can occur as a consequence of changes to risk, effort *or* reward; not necessarily all three. That is, sometimes a new exploitation, tool or method will be chosen because it makes the crime commission process easier, not because it increases the reward (and vice-versa). Considering crime CMO combinations from the offender exploitation viewpoint, the emphasis is on exploiting the most successful mechanisms within a given context as that internal environment shifts.

Historically, to steal vehicles in the UK, offenders were known to use a long pole with a hook or a magnet to reach through a letterbox to lift car keys from inside the home (Copes & Cherbonneau, 2006; Levesley et al., 2004). As technology evolved and keyless entry systems were introduced, a new exploitable opportunity emerged within the same environment, one that was more accessible, lower risk, and less effortful than fishing inside letterboxes. Offenders exploited the change using easily obtainable and useable electronic relay devices to capture wireless key fob signals, unlocking and starting cars without ever touching the keys, reducing the time and effort required whilst minimising the risk of detection (Francillon et al., 2011). Whilst the criminal objective remained the same, the change in technology generated a new exploitable opportunity within the same environment, better satisfying the offender’s success criteria across risk, effort, and reward simultaneously (Clarke & Cornish, 1985).

A further example is online phishing, which involves eliciting an individual’s confidential information (e.g. passwords or banking details) through deception (Lastdrager, 2014; Maggi, 2010). A historic example is the AOHell program, developed in 1995 to automate the process of obtaining password and credit card details from AOL users through the creation of anonymous accounts and messaging (Rekouche, 2011). Phishing techniques subsequently evolved from email-centric attacks to include the use of spoofed websites which often appear more convincing (than generic phishing emails) to victims and thus a more exploitable opportunity (O’Sullivan, 2003). However, the email medium has since seen a renaissance with the emergence of spear-phishing; targeted messages directed at specific victims that substantially increased the probability of elicitation (Wang et al., 2012). Whilst the criminal objective remained unchanged, each progression was driven by new tools or methods generating new exploitable opportunities within the same context, tipping the balance of the rational choice equation in favour of the offender by reducing perceived risks and required effort whilst increasing the likely or realised rewards. For both the vehicular and fraud examples, the resulting change in MO is what provides the empirical trace of that opportunity evolution.

In scanning for *future* utility-motivated opportunities, the focus should therefore be on monitoring changes in a given internal environment – emerging tools, technologies, and methods – that could generate new ways of exploiting existing contexts. This is perhaps the most technology-sensitive of the four SUPA mechanisms, and HS here means watching for innovations that could lower barriers to exploitation, increase scale, or reduce risk for offenders, before those opportunities are realised. There are numerous examples of such threats, many of which are researched by the Dawes Centre of Future Crime at UCL. For example, Internet connected devices can be hacked to undertake mass Denial of Service attacks, autonomous vehicles could be used in ram-raiding attacks or to disrupt energy grids (Brown et al., 2025) or drones can be used by hostile state actors to undertake espionage (Ekblom, 2022). The crime opportunities associated with this type of evolution might not be exploited at all, or not exploited immediately, but they should nevertheless be anticipated and “designed out”.

### Pressure-driven (peripatric)

A sudden change in the current environment, either through the introduction of crime prevention measures, new legislation, or significant external events, can force offenders into (sudden or gradual) isolation from an existing opportunity context, or push exploitation toward new ones. Where this forced severance from an existing opportunity context occurs, new crime opportunities emerge in contexts that would not otherwise have been exploited – this is the pressure-driven mechanism. Where offenders are able to adapt to such pressure, they will be forced over time to innovate and adjust their methods of exploitation accordingly (Ekblom, 1997). Considering offender CMO combinations, this can involve changes to offender contexts, their mechanisms or both: whatever is necessary for continued crime outcome successes.

The pressure-driven mechanism can be related to the notion of displacement which can include tactical, temporal, spatial, offender and target changes (Guerette & Bowers, 2009; Reppetto, 1976). Each form of displacement represents a shift in the opportunity context being exploited rather than simply a change in method. Clarke and Bowers (2017) posit that displacement is an offender level process, whilst adaptation is a population level characteristic. As we are agnostic to who the exploiting offenders are, we can also include offender replacement (Barr & Pease, 1990; Guerette & Bowers, 2009) as a forced change in the environment which can open new opportunities to different groups of offenders with different criminal skill sets (Rossmo & Summers, 2021). In this case, there is still an evolution in the opportunities being exploited (at the aggregate level of all offenders), the identification of which should inform the (current or future) crime prevention enterprise. For example, the introduction of chip and PIN technology removed opportunities that involved the physical use of stolen cards in stores or other locations, pushing exploitation toward opportunities that do not require the PIN, such as online or card-not-present fraud (Finch, 2011).

Another example of pressure-driven opportunity evolution is the cross-border (spatial) and operational displacement of traffickers and sex workers following South Korea’s nationwide anti-trafficking measures (Choo & Rebovich, 2014). Here, prevention measures forced isolation from existing opportunity contexts, with exploitation displaced to new geographic settings. Similarly, a tactical displacement of smuggling has been observed in the Philippines following customs reforms, with offenders declaring goods at lower values or importing them through regions exempt from the reforms (Yang, 2008, 2004).

A further example of environmental change, but due to the ’shock’ of new legislation, is that of prohibited substances. In 1928, the United Kingdom (UK) introduced laws restricting the availability of cannabis (Manning, 2013), gradually constraining the existing opportunity context. This pressure generated new exploitable opportunities in the form of synthetic cannabinoids, which produce similar effects but legally and with greater harm (Cohen & Weinstein, 2018).^4^ In response, the Uk Government (2016) introduced the Psychoactive Substances Act, to ban previously so-called ’legal highs’ and synthetic cannabinoids; itself a further pressure-driven change, with concerns already raised about exploitation shifting toward different, potentially more dangerous substances (Hammersley, 2010). This example illustrates how pressure-driven evolution can cascade: each intervention generates new opportunity contexts, which may in turn require further preventative responses.

Whilst the pressure-driven mechanism was initially focused on a change in the environment through the introduction of crime prevention measures or new legislation, these should not be seen as exclusive instigators. The global Covid-19 pandemic temporarily transformed the opportunity landscape, forcing isolation from physical crime contexts whilst simultaneously opening new exploitable opportunities including new forms of cybercrime (Farrell, 2020; Johnson & Nikolovska, 2024) and the counterfeiting and fraudulent sale of personal protective equipment (PPE) (Kennedy et al., 2021).^5^

Thus, in terms of scanning for *future* pressure-driven opportunities, it is important to consider not only what existing opportunity contexts might be blocked, but what new contexts such blocking might displace or push exploitation toward. Such changes can arise through (1) effective post-hoc reactive crime prevention strategies, (2) new legislation, regulation or laws, or (3) significant external events including pandemics, natural disasters or war. The PESTLE framework of change (political, economic, societal, technological, legal and environmental) provides a more comprehensive range of dimensions through which pressure-driven opportunity evolution might be anticipated. Good futures thinking should consider both the immediate displacement possibilities brought on by sudden drivers of change and the longer-term cascading effects that sustained pressure on an opportunity context may produce.

### Attraction-led (parapatric)

Whereas pressure-driven evolution involves a forced severance from an existing opportunity context, attraction-led evolution epitomises voluntary migration into an adjacent opportunity context, as (existing or new) offenders elect to operate in a new environment. It could be argued that this mechanism encompasses traits of the other three. For example, it aligns closely with the utility-motivated mechanism and the notion that offenders look to reduce perceived risk and effort whilst maximising potential rewards since it makes little sense for an offender to change their operating environment otherwise. However, its key distinction is that the origin of evolution is entirely voluntary and that there is a *measurable change into an adjacent opportunity context* rather than within an existing one. In terms of offender crime CMO combinations, this will be a definite change to context and potentially also a change to mechanism which offers an advantage to the offending population in the aggregate (but perhaps not to individual offenders). As in the utility-motivated evolution case, the advantage might not necessarily be related to the ‘bettering’ of the outcome itself, but a reduction in effort, the risk of detection, or an increase in the scale of the successful outcome.

An example of attraction-led opportunity evolution is the voluntary transition from physical exchanges to cyber markets for the sale of illicit substances, services, information, and objects (Martin, 2014). This resembles utility-motivated elements as it has the potential to reduce the risk taken by an offender (Červený & van Ours, 2019) and is therefore adaptation through choice, but the new opportunity context is located within a *different environment*, a defining feature of the attraction-led mechanism that distinguishes it from utility-motivated evolution.

In horizon scanning for future attraction-led opportunities, these types of voluntary evolutions might be some of the most difficult to anticipate, because they are not as ‘bounded’ by changes in the environment in the way that space-driven or pressure-driven opportunities are. They are, however, likely to conform to the rule of ‘least effort’. In many cases, the switch will be to cyber versions of physical criminal acts, including online fraud (Kamps & Kleinberg, 2018; Trozze et al., 2022), cyber-physical attacks (Gómez-Quintero et al., 2024), and online sexual exploitation (Wortley et al., 2024). A clear example is child sexual abuse material (CSAM), which, having been in decline, rapidly proliferated with the advent of the internet, illustrating how offenders voluntarily migrating toward a new opportunity context can amplify the reach and ease of certain crimes through utilising digital technologies (Wortley & Smallbone, 2012).

However, attraction-led opportunity evolution need not always move in the direction of cyber contexts. Whilst previous research has documented attackers gaining access to cryptocurrency wallets through purely online methods, such as technical support scams (Acharya et al., 2024), recent incidents demonstrate that attackers have voluntarily migrated toward the physical realm. Wrench attacks, for example, involve the physical threat or use of violence (perhaps using a wrench) and coercion to steal – or gain access to – people’s cryptocurrency wallets (Ordekian et al., 2024). These attacks, which require little technical sophistication, allow perpetrators to bypass the robust digital security measures that were originally designed to protect assets in online spaces, representing a voluntary migration into an adjacent physical opportunity context where those protections do not apply. At the time of writing (November 2025), one such incident resulted in the theft of $11 million (Sigalos, 2025).

### Evolutionary pathways of crime opportunity

It is important to highlight that the evolutionary pathway of a crime opportunity can involve multiple SUPA mechanisms. Our previous vehicle theft example provides a clear illustration: the introduction of electronic immobilisers represented a pressure-driven evolution, forcing offenders into isolation from an existing opportunity context – stealing cars directly – and toward a new one: stealing the keys through letterboxes to bypass security measures (Copes & Cherbonneau, 2006). As keyless entry systems were subsequently introduced, a utility-motivated evolution occurred as a new exploitable opportunity emerged within the same context – offenders developed relay attacks, using devices to capture and relay wireless key fob signals to unlock and start cars without ever touching the keys (Francillon et al., 2011).

Similarly, fraudulent deception demonstrates multiple SUPA mechanisms: more primitive in-person attempts such as charity scams (where someone pretends to be a charity representative, asking for donations but keeping the money) migrated to the online environment in an attraction-led evolution, as offenders voluntarily moved into an adjacent opportunity context offering greater scale and reduced risk. This was followed by utility-motivated evolution within the online space, as new exploitable opportunities emerged in the form of distinct fraud types such as phishing, romance scams, and advance-fee fraud. These examples demonstrate how a single crime type can traverse multiple SUPA mechanisms as offenders continuously respond to new constraints, new tools, and new contexts.

## Discussion

This paper has aimed to reconcile concepts from crime opportunity evolution with parallel ideas of adaptation in biological species evolution. Our intention has been to produce a framework to categorise different *types* of crime opportunity evolution, and to identify the conditions under which each type arises. Whilst new crime types rarely materialise – since the crime type seldom changes, only the environmental opportunities through which it is pursued and the methods by which those opportunities are exploited – it is precisely this evolution that the SUPA framework is designed to capture. The MO recording system established by Atcherley (1913) and modernised by National Policing Improvement Agency (2012) – which likely has crime recording equivalents in non-UK contexts – provides the practical measurement infrastructure for this: where new crime opportunities are realised as crime, changes in MO attributes provide the empirical trace through which that evolution can be measured. Detecting meaningful opportunity evolution through MO analysis requires distinguishing sustainable change from short-lived adaptation, and as we proposed earlier, *novelty is only relevant if it is sustainable*. Horizon scanning for such changes, using MO recording systems as a signal of emerging opportunity evolution, provides a practical "early warning" mechanism through which law enforcement and prevention practitioners can anticipate changes that may occur at scale rather than merely react to crime opportunity change. As Eck et al. (2026) note, crime opportunity has two elements (creation and exploitation) and realised crime is a subset of these opportunities, and MO is how that realisation becomes visible.

Offender populations that adapt their exploitation of available opportunities are those that survive (or emerge) in a never-ending arms race with those who seek to prevent crime (Ekblom, 2001). To better describe and categorise how crime opportunities evolve, we conceptualised the four SUPA mechanisms, each describing a distinct pathway through which new crime opportunities emerge and are realised through changes in MO. We have demonstrated that many historic evolutions and future threats can be neatly categorised into (at least) one of these evolutionary mechanisms. Understanding which SUPA mechanism is driving opportunity change should help preventative focus: a space-driven opportunity may require context-specific design interventions; a utility-motivated one calls for monitoring the internal technological landscape and designing out emerging tools; a pressure-driven one demands anticipating where blocked exploitation will displace to; and an attraction-led one requires identifying what is drawing offenders voluntarily into new contexts before that migration becomes established. In each case, identifying the mechanism early should increase the likelihood of intervening (cost-) effectively before opportunities become established and are harder to disrupt. Together, the SUPA mechanisms provide a framework for thinking about and anticipating future crime opportunity.

Blocking and restricting exploitation might be best achieved through law and regulation, codes of practice, building and product design, or retrofitting and opportunity withdrawal. In cases where environmental change is not planned or easily anticipated/controlled (e.g. pandemics and natural disasters), the ability to block or restrict through policy and other options may be more limited. Here, foresight informed by the SUPA framework can play an important role: by anticipating which mechanism is likely to be activated and what opportunity contexts may emerge, practitioners can prepare mitigation strategies in advance, reducing the time between opportunity emergence and an effective preventative response. Where opportunities are realised as crime before prevention measures can be put in place, foresight can equally inform approaches to harm mitigation by reducing the scale, duration, or impact of exploitation rather than preventing it entirely. It is also worth noting that pressure on an opportunity context does not inevitably produce displacement; Walsh (1994) documents how crime forms can become entirely obsolete when the conditions that sustain them disappear, suggesting that extinction of an opportunity, rather than its displacement, is a possible outcome that the SUPA framework should account for.

From a preventative perspective, it would also be beneficial to undertake a mapping exercise of known opportunity evolutions and analyse the degree to which each of the 5 Situational Crime Prevention (SCP) mechanisms (Cornish & Clarke, 2003; Johnson, 2024) may have been effective at countering each of the SUPA mechanisms. This could advance understanding of whether SCP interventions prevent or merely temporarily displace opportunity evolution, and how the type of SUPA mechanism shapes the appropriate preventative response. Further exploration of opportunity evolution mechanisms by type of crime would also be valuable for crime-specific future-orientated prevention planning. The empirical grounding of such insights will depend on the development of systems that enable the automated extraction of MO attributes from free-text crime records. Without the ability to record or extract MO attributes at scale, we cannot detect the small but persistent deviations that signal emerging opportunity evolution. Once opportunity evolution is better measured empirically through its MO trace, we can begin to explore the prevalence of different SUPA mechanisms across crime types and develop intelligent systems that connect emerging opportunity evolution to pre-emptive preventative solutions.

As with any framework, there are limitations to the approach taken here. We have adapted a specific type of evolutionary framework (biological) for the consideration of future crime and future opportunities, and important ideas in the principles of cultural evolution laid out by Boyd and Richerson (1988) are also relevant to crime evolution. However, we based our classification on the biological speciation framework because it fits the role of the environment in evolution. The importance of the environment in the opportunity theories of crime is precisely what distinguishes them from other types of crime theory and gives them a clear and practical focus.

However, as noted by Ekblom (2017) opportunities are not purely environmental but emerge from an interaction between the environment and offender resources – including tools, skills, and knowledge – and goals. Whilst the SUPA mechanisms describe the conditions under which new opportunity contexts arise, it is this interaction between context and offender resources that ultimately determines whether a potential opportunity is realised as crime. A new opportunity context may exist but remain unexploited if offenders lack the resources or goals necessary to take advantage of it; conversely, the acquisition of new resources – whether through learning, communication, or access to new tools – can activate opportunities that have existed but gone unrealised. In this sense, offender resources and goals are not merely background conditions but active determinants of when and how crime opportunity evolution becomes visible through changes in MO. Returning to the language of Cornish (1989) the ’choice structuring properties’ of crimes are multi-faceted and are interactions between decision makers and their environments.

A specific question regarding within-offender crime-specific adaptation is whether they move from physical to virtual environments (or, indeed, between them). This is contested by some academic research which cites the differing skill sets required of offenders (Farrell & Birks, 2018). Nonetheless, Farrell and Birks (2018) propose that a subset of offenders might undertake "adaptive switching" if they recognise new opportunities and attain the skills and necessary resources to voluntarily operate in a new (cyber) environment. The advent of Cybercrime-as-a-Service (Collier et al., 2022) which lowers the barrier to entry for offending, might further encourage adaptive switching among existing offenders whilst also drawing new ones into contexts previously beyond their capability, but this is perhaps most likely over longer timescales. In SUPA terms, this kind of within-offender migration is most closely associated with attraction-led evolution, where the appeal of an adjacent context draws voluntary movement, though the reduced barrier to entry also has utility-motivated characteristics.

A related but distinct phenomenon is that of hybrid exploitation, where offenders do not fully migrate between environments but instead deliberately straddle two adjacent contexts within a single criminal episode. This is only possible where an attractive alternative environment is accessible and can be exploited in combination with the original environment. The wrench attack example discussed earlier illustrates this well (Ordekian et al., 2024; Sigalos, 2025): the target asset (a cryptocurrency wallet) exists in the digital environment, but exploitation occurs in the physical one, precisely because the physical context bypasses the digital security measures that would otherwise prevent access. These hybrid crimes complicate HS because they may not be visible within any single environment, requiring analysts to consider interactions between adjacent contexts.

The discussion of how offenders navigate and exploit adjacent environments – whether through sequential migration or hybrid exploitation within a single episode – raises a broader point about the relationship between displacement and adaptation that is worth addressing directly. Displacement is a well-established concept in criminology and is most closely captured by the pressure-driven SUPA mechanism. However, displacement alone is insufficient for understanding how crime opportunities evolve: it is inherently reactive and does not account for the full range of pathways through which new crime opportunities emerge. Adaptation, as captured by the SUPA framework, is a wider concept: it encompasses not only forced changes such as displacement (pressure-driven) but also opportunity evolution that arises through environmental differentiation (space-driven), internal innovation within a stable context (utility-motivated), and voluntary migration into adjacent contexts (attraction-led) – the latter of which, as illustrated by adaptive switching, can occur independently of any prevention-driven pressure. In this sense, displacement is best understood as one possible manifestation of adaptation captured by the SUPA framework, which provides a richer conceptual vocabulary for horizon scanning and prevention planning than displacement alone.

Together, by considering evolution in offender populations (both within and between potential offenders), these considerations demonstrate that the SUPA framework can account for the cultural adaptations driven by learning and communication, adding a dynamic human dimension to what might otherwise be a purely environmental classification. Both utility-motivated and attraction-led evolution are less bounded by environmental conditions and demonstrate more scope for ‘trial and error’. These are perhaps the SUPA mechanisms most likely to be accelerated through individual offender learning and communication networks. To some extent, this makes them more difficult to anticipate, which further makes the case for the need for systematic approaches to HS that monitor not just environmental change but the internal innovation and migration dynamics of offending populations.

In conclusion, we have explored how and why crime opportunities evolve and proposed that different types of evolution occur under different environmental conditions. Focusing on the *evolution of crime opportunity*, rather than attempting the often infeasible task of identifying entirely new crimes, provides a more practical and actionable approach to horizon scanning and prevention. The ideas discussed here establish the SUPA framework for thinking about future crime opportunity, helping to organise an analyst’s thinking and encourage a more systematic consideration of the different dimensions of a problem that might otherwise receive little attention or be overlooked.

## Appendix A Modus operandi attributes

See Table 1.

**Table 1:** MO attributes according to the National Policing Improvement Agency and Atcherley’s original classification

STOP CRIMES, national policing improvement agency	Atcherley’s original MO classification system	Description based on national policing improvement agency (2012)
Style	Style	Any method(s) of deception used. For example: • Dressed as a church minister • Documents claiming to be an insurance collector
Time	Time	Time relative to an event (not to the clock). For example: • After the occupant had left for work • On a market day • On an early closing day • Whilst a specific event was running
Objective	Object	The objective of the crime. For example: • Gain • Lust • Revenge
Pal	Pal	Accomplice or friend?
Class	Classword	The target of the crime. For example: • People • Places • Property • Services
Reason	Tale	Reason for being in the area, the criminal’s self-account, their history, etc
Instrument	Means	Instrument used to commit the crime. For example: • Crowbar • Unusual bodily force (e.g., sitting on victim)
Mode	Transport	Mode of transport used to commit the crime
Entry	Entry	The actual point where entry was made
Signature	Trademark	Anything unusual done by the criminal, their ‘signature’. For example: • Creates an escape route by wedging doors open • Closes curtains • Ejaculates into women’s underwear • Defecates

## Footnotes

1 At the time of writing, in the UK, measures included in the Data (use and access) Bill would make it illegal to create such content.

2 The NPIA was later replaced by the College of Policing.

3 Eck et al. (2026) also draw a parallel between opportunity creation and exploitation and the concepts of ’occasions for action’ and ’temptations’ introduced by Mayhew et al. (1976), demonstrating that the distinction between the birth of a crime opportunity and the occasion of its exploitation was present within that foundational paper from the outset.

4 The lengthy interval between the 1928 legislation and the emergence of synthetic cannabinoids illustrates how pressure-driven displacement can be latent, materialising only when enabling conditions – in this case, advances in synthetic chemistry – make new opportunity contexts accessible.

5 Interestingly, Covid-19 adaptations demonstrated more immediate pressure-driven evolution than many of the other examples discussed, illustrating that the conditions under which isolation from an existing opportunity context occurs can determine whether the resulting displacement is gradual or more of a step change.
